# Thermodynamic Assessment of the Pyrazinamide Dissolution Process in Some Organic Solvents

**DOI:** 10.3390/molecules29215089

**Published:** 2024-10-28

**Authors:** Jesus Tovar-Amézquita, Cristian Rincón-Guio, Francy Elaine Torres-Suarez, Magda Melissa Florez, Claudia Patricia Ortiz, Fleming Martinez, Daniel Ricardo Delgado

**Affiliations:** 1 Sitafi Group Ingeniería S.A.A., Palermo 412001, Huila, Colombia; jesustovaramezquita@gmail.com; 2 Rectoría Virtual, Ingeniería Industrial, Corporación Universitaria Minuto de Dios-UNIMINUTO, Bogotá 110321, Cundinamarca, Colombia; cristian.rincon.g@uniminuto.edu; 3 Programa de Medicina, Grupo de Investigación CIST-Centro de Investigación en Salud Para el Trópico, Universidad Cooperativa de Colombia, Sede Santa Marta, Troncal del Caribe, Mamatoco, Santa Marta 470001, Magdalena, Colombia; francy.torress@campusucc.edu.co (F.E.T.-S.); magda.melissa@campusucc.edu.co (M.M.F.); 4 Programa de Administración en Seguridad y Salud en el Trabajo, Grupo de Investigación en Seguridad y Salud en el Trabajo, Corporación Universitaria Minuto de Dios-UNIMINUTO, Neiva 410001, Huila, Colombia; claudia.ortiz.de@uniminuto.edu.co; 5 Grupo de Investigaciones Farmacéutico-Fisicoquímicas, Departamento de Farmacia, Facultad de Ciencias, Universidad Nacional de Colombia, Sede Bogotá, Carrera 30 No. 45-03, Bogotá 110321, Cundinamarca, Colombia; fmartinezr@unal.edu.co; 6 Programa de Ingeniería Civil, Grupo de Investigación de Ingenierías UCC-Neiva, Facultad de Ingeniería, Universidad Cooperativa de Colombia, Sede Neiva, Calle 11 No. 1-51, Neiva 410001, Huila, Colombia

**Keywords:** pyrazinamide, solubility, tuberculosis, shake-flask method, thermodynamic properties, Hoftyzer-van Krevelen, Fedor, solubility parameters

## Abstract

Pyrazinamide is a first line drug used for the treatment of tuberculosis, a pathology that caused the death of more than 1.3 million people in the world during 2022, according to WHO, being a drug of current interest due to its relevance in pharmaceutical and medical sciences. In this context, solubility is one of the most important physicochemical parameters in the development and/or optimization of new pharmaceutical forms, so the present work aims to present a thermodynamic study of the solubility of pyrazinamide in nine organic solvents of pharmaceutical interest. Using the shake-flask method and UV/Vis spectrophotometry, the solubility of this drug was determined at 9 temperatures; the maximum solubility was obtained in dimethyl sulfoxide at 318.15 K ($x_{2}=0.0816\pm 0.004$) and the minimum in cyclohexane at 283.15 K ($1.73\pm 0.05\times 10^{-5}$). From the apparent solubility data, the thermodynamic functions of solution and mixing were calculated, indicating an endothermic process. In addition, the solubility parameter of pyrazinamide was calculated using the Hoftyzer-van Krevelen (32.90 MPa^1/2^) and Bustamante (28.14 MPa^1/2^) methods. The maximum solubility was reached in dimethyl sulfoxide and the minimum in cyclohexane. As for the thermodynamic functions, the entropy drives the solution process in all cases. In relation to the solubility parameter, it can be analyzed that the mathematical models offer approximations; however, the experimental data are still primordial at the time of inferring any process.

## 1. Introduction

**Figure 1:**
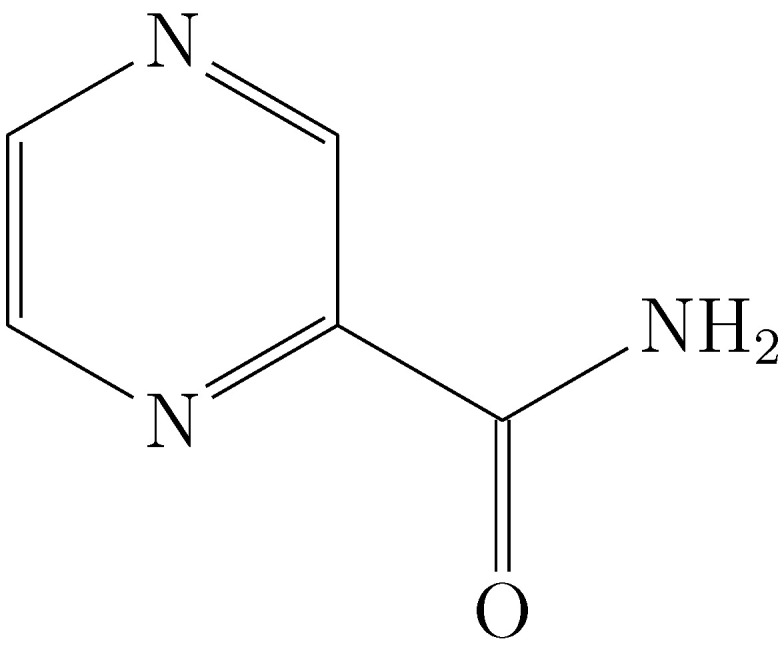
Molecular structure of the pyrazinamide [5].

Pyrazinamide (pyrazine-2-carboxamide (Figure 1)) is a nicotinamide analog administered as a first-line treatment for patients with tuberculosis caused by multidrug-resistant tuberculosis. This drug acts as a prodrug because its reduced form (pyrazinoic acid) is responsible for its antibacterial activity [1]. Although the complete mechanism of action is unknown, it has been described that this drug accumulates in the bacteria, creating an acidic environment and inhibiting enzymes involved in the DNA transduction process [2,3]. The drug’s behavior in the presence of acid and its chemical conformation give it advantages in efficacy for pulmonary TB due to its ability to cross cell membranes [4].

One of the drawbacks of the massive use of pyrazinamide in the treatment of tuberculosis is that part of the drug supplied to the patient is not metabolized and becomes an emerging contaminant; thus, the NORMAN network classifies pyrazinamide as a Level 2 emerging contaminant [6].

The above shows that nowadays, in addition to offering effective pharmaceutical dosage forms, factors such as environmental impact must also be taken into account, which may be related to the design of more efficient processes and/or pharmaceutical dosage forms [7,8].

Solubility is one of the most important physicochemical properties involved in the understanding of biological processes such as pharmacodynamics and pharmacokinetics, and in industrial processes such as synthesis, purification, recrystallization, quality analysis, preformulation, and formulation, among others [9,10,11,12]. In addition, solubility is also related to treatment processes for contaminated water and soil [8], and environmental impact assessments [13]. In this context, given the importance of solubility, one of the most important lines of research is the development of models to predict drug solubility in order to accelerate development processes and optimize industrial processes [14,15,16]. Therefore, it is clear that experimental data must be generated with methods that guarantee maximum data quality, and given that predictive models are currently only partially effective, experimental data generation is one of the most relevant lines of research today [17,18].

In relation to pyrazinamide, some solubility studies have been reported in cosolvent mixtures and pure solvents at different temperatures; however, the number of investigations is limited, so the development of new research related to the solubility of this drug allows, in addition to expanding the information already reported, rationalize and optimize processes; therefore, understanding the possible molecular phenomena involved in the solution process is one of the most booming lines of research in the field of pharmaceutical sciences [19,20,21,22,23].

Therefore, it is of great importance to develop new research on solvents of pharmaceutical interest, such as low-toxicity alcohols used in formulation and/or design, low-polarity solvents used in quantification methods, and some semi-polar solvents with high solvent power.

Accordingly, in this work, the solubility of pyrazinamide in ethanol, 1-propanol, butanol, 1-octanol, 1,4-dioxane, cyclohexane, chloroform, *N*,*N*-dimethylformamide (DMF), dimethylsulfoxide (DMSO), and *N*-methyl-2-pyrrolidone (NMP), nine solvents of industrial and pharmacological interest, is presented [24,25,26,27].

In addition, the thermodynamic analysis of the solubility of pyrazinamide is reported and some mathematical models of interest to the pharmaceutical industry for predicting the solubility of the drug under study are evaluated, given the importance that this line of research has recovered in order to optimize industrial processes and ultimately make them more environmentally sustainable [28,29].

## 2. Results

### Experimental Solubility ($x_{3}$)

Table 1 presents the mole fraction of pyrazinamide in 9 pure solvents at different temperatures (278.15 K–318.15 K). In all cases, the solubility of the drug increases with increasing temperature of the system, indicating an endothermic process.

**Figure 2:**
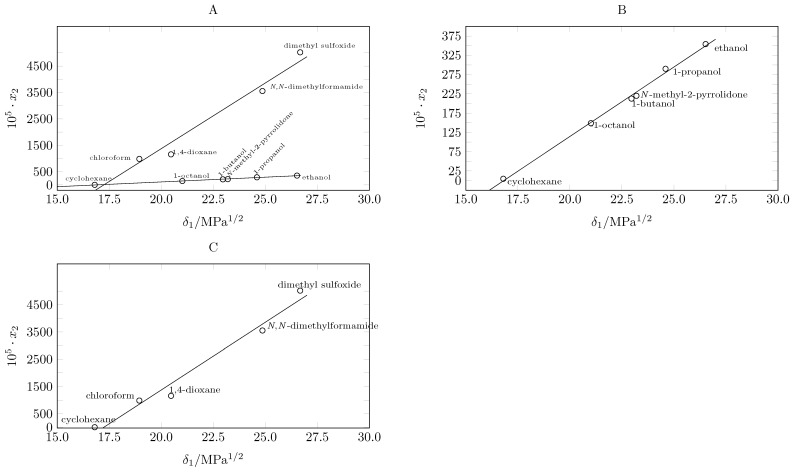
Correlation between the solubility of pyrazinamide at 298.15 K and the solubility parameter of organic solvents.

The maximum solubility of pyrazinamide is reached in dimethyl sulfoxide at 318.15 K and the lowest in cyclohexane at 283.15 K, which agrees with Hildebrand’s theory [10,30], reaching its maximum solubility in the solvent with the solubility parameter closest to that of pyrazinamide (35.10 MPa^1/2^ [31]). In this case, it is dimethyl sulfoxide, whose solubility parameter is 26.7 MPa^1/2^ [32]. When evaluating the solubility behavior of pyrazinamide in the 9 solvents as a function of the solubility parameter, two trends emerge (Figure 2A).

The first trend is observed when plotting the solubility of pyrazinamide at 298.15 K as a function of the solubility parameter. An almost linear behavior is presented when relating the solubility data in cyclohexane (16.8 MPa^1/2^ [32]), 1-octanol (21.0 MPa^1/2^ [32]), *N*-Methyl-2-pyrrolidone (23.0 MPa^1/2^ [32]), 1-butanol (23.2 MPa^1/2^ [32]), and 1-propanol (24.6 MPa^1/2^ [32]); the linearity of the solubility in alcohols is highlighted, which increases as the solubility parameter of the alcohol increases (Figure 2B).

A second trend is shown by plotting. When graphing the solubility as a function of the solubility parameter of cyclohexane (16.8 MPa^1/2^ [32]), chloroform (18.9 MPa^1/2^ [32]), 1,4-dioxane (20.5 MPa^1/2^ [32]), *N*,*N*-dimethylformamide (24.9 MPa^1/2^ [32]), and dimethylsulfoxide (26.7 MPa^1/2^ [32]), a linear trend is also observed (Figure 2C). Although, in both cases, a direct relationship can be observed between the solubility of pyrazinamide and the polarity of the solvents, it is clear that the molecular structure of the solvents has a significant influence on the magnitude of the solubility.

**Figure 3:**
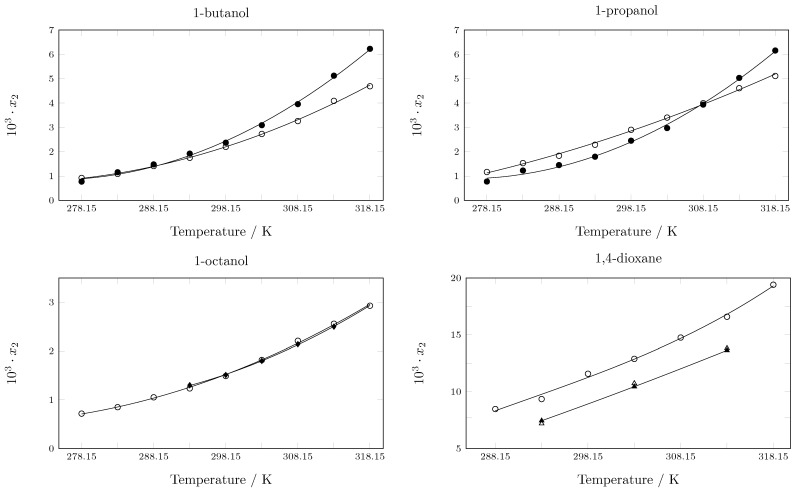
Experimental solubility reported in this work and by other authors in various pure solvents. ∘: In this work; •: Zhang et al. [37]; ⧫: Blokhina et al. [36]; ▲: Maharana and Sarkar [33]; ∆: Hermanto et al. [34].

When comparing the results of this work with some data reported in the literature, Maharana and Sarkar [33] report the solubility of pyrazinamide in 1,4-dioxane at 293.15 K, 303.15 K, and 313.15 K, and the difference is 10–30%. Hermanto et al. also reported solubility data in 1,4-dioxane at the same temperature, presenting differences between 21–25% [34]. On the other hand, Xi et al. [35] report the solubility of pyrazinamide in 1-propanol from 278.15 to 318.15 K: at low temperatures (278.15–298.15 K), the difference is approximately 30%; between 303.15 and 308.15 K, the difference is approximately 5%; and at other temperatures, the difference is approximately 15%. Blokhina et al. report values in 1-Octanol at 293.15, 298.15, 303.15, 308.15, and 313.15 K [36]. In all cases, the differences are less than 5%. Finally, Zhang et al. report solubility data in 1-propanol and 1-butanol [37], and the discrepancies between the data are between 15–20% (Figure 3). This discrepancy between experimental data of a substance in the same solvents under similar conditions can be a consequence of factors such as: equilibrium time, purity of the reagents, uncertainty in the measurement, and experimental conditions, among others. This discrepancy between solubility data obtained by different research groups is common and does not imply that one or the other data are better, but ensures the quality of the data is important to strictly report the methodology used.

A factor that can intervene in the change in solubility of a drug are polymorphic changes or formation of solvates [38,39,40]. There is a need for improvement; therefore, it is necessary to evaluate whether, in addition to factors such as temperature and solvent, the changes in the solubility of pyrazinamide are also a consequence of some change in its crystalline structure, or the formation of a complex that increases or decreases the solubility of the drug.

Thus, a sample of the drug in equilibrium with the saturated solution (in each of the solvents studied) was taken and analyzed by differential scanning calorimeters (Table 2, Figure 3), except for the original sample of pyrazinamide, which presents a clear polymorphic transition at 422.0 K. According to the data reported by Cherukuvada et al. [41], the original sample possibly corresponds to the α form, since two peaks corresponding to the polymorphic transition and the melting point are observed; as for the equilibrium samples, the DSC analysis does not clearly show the peak corresponding to the polymorphic change in the α form, and it is therefore inferred that the samples correspond to the γ form of pyrazinamide.

Regarding the α form, the data of $T_{\mathrm{trs}}$, $\Delta_{\mathrm{trs}}H$, $T_{\mathrm{fus}}$, and $\Delta_{\mathrm{trs}}H$ agree with those reported by Negoro et al. [42], Castro et al. [43], Baaklini et al. [44], Maharana and Sarkar [33], and Cherukuvada et al. [41].

On the other hand, data from the solid phases in equilibrium with each of the saturated solutions of the different organic solvents agree with the results reported by Cherukuvada et al. [41] for the γ form of pyrazinamide.

## 3. Thermodynamic Functions

From the experimental solubility data of pyrazinamide in pure solvents at the temperatures studied, the apparent thermodynamic functions of solution and mixture are calculated.

### 3.1. Thermodynamic Functions of Solution

**Figure 4:**
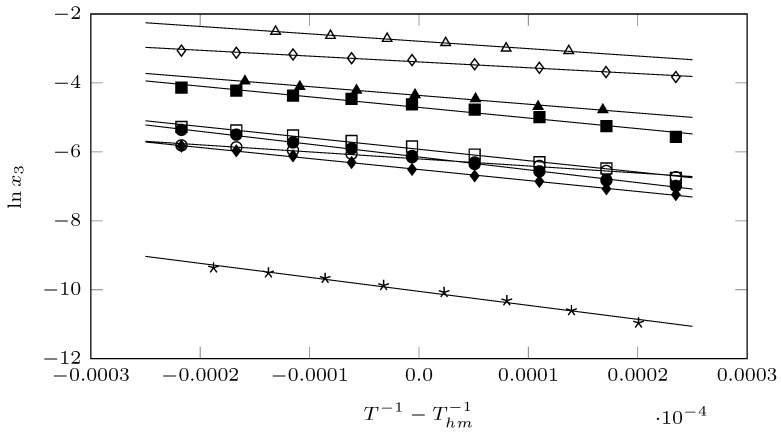
van’t Hoff–Krug plot for the solubility of pyrazinamide (3) in different organic solvents (⋆: cyclohexane; ⋄: DMF; ▲: 1,4-dioxane; ∆: DMSO; ■: chloroform; □: 1-propanol; ◯: NMP; •: 1-butanol, and ⧫: 1-octanol.

The thermodynamic solution functions were calculated according to the van’t Hoff–Krug model. This model is developed by introducing the harmonic temperature term ($T_{\mathrm{hm}}$) into the van Hoff equation. According to Krug et al., this process allows us to reduce the propagation of uncertainties, which can affect the accuracy [45,46,47,48]:

(1) $$\Delta_{\mathrm{soln}}H^{o}=-R{\left[\frac{𝜕\ln x_{2}}{𝜕\left(T^{-1}-T_{\mathrm{hm}}^{-1}\right)}\right]}_{p}$$

(2) $$T_{hm}=\frac{n}{\sum_{i=n}^{n}1/T}$$

(3) $$\Delta_{\mathrm{soln}}G^{o}=-RT_{\mathrm{hm}}.\mathrm{intercept}$$

(4) $$T_{\mathrm{hm}}\Delta_{\mathrm{soln}}S^{o}=\left(\Delta_{\mathrm{soln}}H^{o}-\Delta_{\mathrm{soln}}G^{o}\right)$$

where $\Delta_{\mathrm{soln}}H^{o}$ is the enthalpy of solution (in kJ·mol^−1^), *R* is the gas constant (in kJ·mol^−1^), *T* is the study temperatures (in K), $T_{\mathrm{hm}}$ is the mean harmonic temperature (in K), where *n* is the number of temperatures studied in each of the solvents. $\Delta_{\mathrm{soln}}G^{o}$ is the Gibbs energy of solution (in kJ·mol^−1^), and $T\Delta_{\mathrm{soln}}S^{o}$ is the component related to the entropy of solution (in kJ/mol^−1^). In Equation (3), the intercept corresponds to the value of *b* of the respective linear equation (y=am+b; $\ln x_{2}=m\cdot \left(T^{-1}-T_{\mathrm{hm}}^{-1}\right)+b$) that represents the solubility trend in each of the solvents studied, graphed in Figure 4. So, $b=\ln x_{2}^{T_{\mathrm{hm}}}$.

Table 3 shows the thermodynamic functions of the solution process of pyrazinamide (2) in nine organic solvents. The Gibbs energy is positive in all cases; it is important to clarify that the standard Gibbs energy of solution is not an indicator of spontaneity, its positive value is a consequence of expressing solubility in molar fraction. Therefore, according to Equation (3), which is technically $\Delta_{\mathrm{soln}}G^{o}=-RT_{\mathrm{hm}}\ln x_{2}$, negative values are not obtained in any case. DMSO is identified as the most energetically favorable solvent and cyclohexane as the most unfavorable for the solution process of the pyrazinamide (2).

As for $\Delta_{\mathrm{soln}}H^{o}$, it is positive in all cases, indicating that the solution process is endothermic. As for the organizational term ($T_{\mathrm{hm}}\Delta_{\mathrm{soln}}S^{o}$), positive values are also obtained, indicating an entropic driving of the pyrazinamide solution process in all solvents.

The energetic and organizational contribution to the solution process can be evaluated using Perlovich’s plot. Thus, values located in sectors I, IV, V, and VIII indicate enthalpic conduction and values located in sectors II, III, VI, and VII indicate entropic conduction, in accordance with:

- Sector I: $\Delta_{\mathrm{soln}}H^{o}>T\Delta_{\mathrm{soln}}S^{o}$
- Sector II: $T\Delta_{\mathrm{soln}}S^{o}>\Delta_{\mathrm{soln}}H^{o}$
- Sector III: $\Delta_{\mathrm{soln}}H^{o}<0;T\Delta_{\mathrm{soln}}S^{o}>0;|T\Delta_{\mathrm{soln}}S^{o}\left|>\right|\Delta_{\mathrm{soln}}H^{o}|$
- Sector IV: $\Delta_{\mathrm{soln}}H^{o}<0;T\Delta_{\mathrm{soln}}S^{o}>0;|\Delta_{\mathrm{soln}}H^{o}|>|T\Delta_{\mathrm{soln}}S^{o}|$
- Sector V: $\Delta_{\mathrm{soln}}H^{o}<0;T\Delta_{\mathrm{soln}}S^{o}<0;|\Delta_{\mathrm{soln}}H^{o}|>|T\Delta_{\mathrm{soln}}S^{o}|$
- Sector VI: $\Delta_{\mathrm{soln}}H^{o}<0;T\Delta_{\mathrm{soln}}S^{o}<0;|T\Delta_{\mathrm{soln}}S^{o}\left|>\right|\Delta_{\mathrm{soln}}H^{o}|$
- Sector VII: $\Delta_{\mathrm{soln}}H^{o}>0;T\Delta_{\mathrm{soln}}S^{o}<0;|T\Delta_{\mathrm{soln}}S^{o}\left|>\right|\Delta_{\mathrm{soln}}H^{o}|$
- Sector VIII: $\Delta_{\mathrm{soln}}H^{o}>0;T\Delta_{\mathrm{soln}}S^{o}<0;|\Delta_{\mathrm{soln}}H^{o}|>|T\Delta_{\mathrm{soln}}S^{o}|$

**Figure 5:**
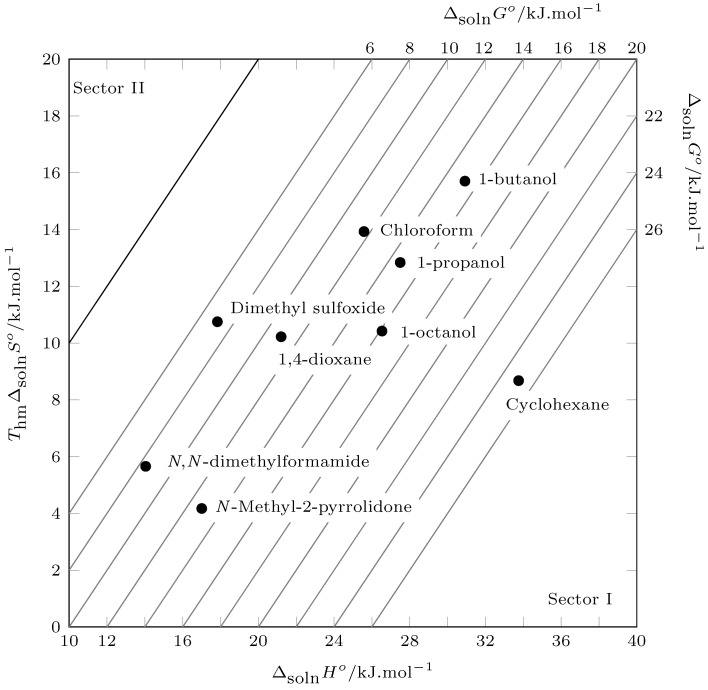
Relation between enthalpy ($\Delta_{\mathrm{soln}}H^{o}$) and entropy ($T_{\mathrm{hm}}\Delta_{\mathrm{soln}}S^{o}$) in terms of the process of pyrazinamide (3) in nine organic solvents at $T_{\mathrm{hm}}$. The isoenergetic curves for $\Delta_{\mathrm{soln}}G^{o}$ are represented by dotted lines.

According to Figure 5, all data are recorded in sector I, which indicates that the solution process in all cases is driven by the enthalpy of solution.

### 3.2. Thermodynamic Functions of Mixing

**Figure 6:**
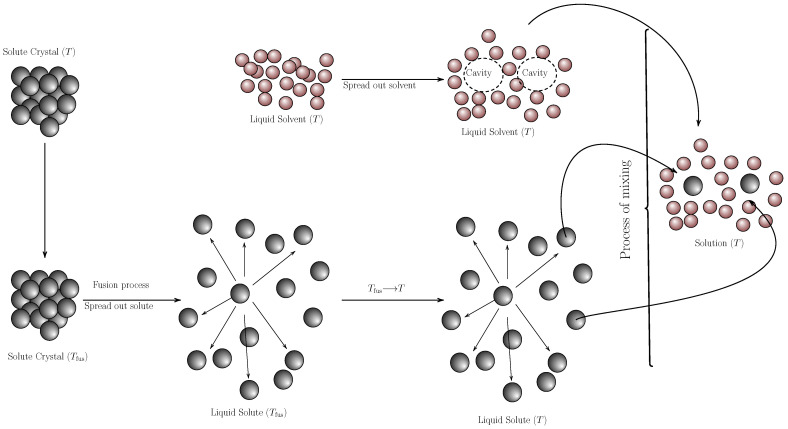
Diagram of the hypothetical solution process [55].

The solution process involves two sub-processes (Figure 6)

1. The hypothetical melting of the solute to sub-cooled liquid and the restructuring of the solvent molecules to form the cavity that will house the solute molecule.
2. Interaction between the solute and solvent molecules (mixing process) to form the solution.

Mathematically, the solution process is expressed as (5):

(5) $$\Delta_{\mathrm{soln}}f^{o}=\Delta_{\mathrm{mix}}f^{o}+\Delta_{\mathrm{fus}}f^{T_{\mathrm{hm}}}$$

Thus, from Equation (5), the component of the mixing process ($\Delta_{\mathrm{mix}}f^{o}$) is cleared:

(6) $$\Delta_{\mathrm{mix}}f^{o}=\Delta_{\mathrm{soln}}f^{o}-\Delta_{\mathrm{fus}}f^{T_{\mathrm{hm}}}$$

As has been described previously in the literature, in this research, the $\Delta_{\mathrm{soln}}f^{o}$ values for the ideal solution processes were used instead of $\Delta_{\mathrm{fus}}f^{\mathrm{hm}}$ [49].

In this case, in addition to the energy involved in the melting process, the energy involved in the polymorphic transition process must be taken into account. Thus, the ideal solubility data, from which the thermodynamic functions of the ideal process are calculated, are calculated as [31]:

(7) $$\ln x_{2}^{id}=-\frac{1}{RT}\left[\Delta_{m}H\left(1-\frac{T}{T_{m}}\right)+\Delta_{\mathrm{trs}}H\left(1-\frac{T}{T_{\mathrm{trs}}}\right)\right]+\frac{\Delta C_{p}}{R}\left(\frac{T_{m}-T}{T}\right)-\frac{\Delta C_{p}}{R}\ln\left(\frac{T_{m}}{T}\right)$$

where *T*, $T_{\mathrm{prt}}$, and $T_{m}$ (in K), $\Delta_{m}H$ and $\Delta_{\mathrm{trs}}H$ are the enthalpy of fusion and phase transition enthalpy (in kJ·mol^−1^) of the solute, *R* is the gas constant (in kJ·mol^−1^ K^−1^), and $\Delta C_{p}$ is the differential heat capacity of fusion (in kJ·K^−1^ mol^−1^) [50]. Some researchers, like Hildebrand and Scott [51], Neau and Flynn [52], Neau et al. [53], and Opperhuizen et al. [54], assume $\Delta C_{p}$ as the entropy of fusion ($\Delta_{m}S$), which is calculated as $\Delta_{m}H/T_{m}$ (the calculations for the ideal process can be found in the Supplementary Materials).

The thermodynamic functions of the mixing process are shown in Table 4. Thus, the highest values of Gibbs energy and enthalpy of mixing are presented in cyclohexane, which can be an indicator of low affinity between the solute and solvent molecules. The value of enthalpy of mixing is related to the process of formation of the cavity to accommodate the solute molecule, so the low solubility in this solvent can be related to this process. The behavior in 1,4-dioxane and chloroform shows the same tendency, but the Gibbs energies of mixing are much lower, indicating a greater affinity between the pyrazinamide molecules and these two solvents. In addition, the enthalpies of mixing also decrease, indicating that the formation of the cavity requires less energy, which, in general, favors the process. In dimethylsulfoxide, *n*-methyl-2-pyrrolidone and dimethylformamide, negative values are obtained for the Gibbs energy of mixing, indicating that, in these solvents, the highest affinity between solute and solvent is present. It should be noted that the standard thermodynamic functions of mixture, as well as the standard thermodynamic functions of solution, are not an indicator of spontaneity, but rather an indicator of affinity. For alcohols (1-propanol, 1-butanol, and 1-octanol), positive Gibbs energies and mixing enthalpies are also presented. For the three alcohols, the affinity between the solute and the solvent is related to the size of the aliphatic chain of the alcohol, with a better affinity between pyrazinamide and the alcohol with the shortest aliphatic chain. Finally, when evaluating the mixing entropy, except for dimethylformamide, it is positive, indicating a preference for the mixing process.

**Figure 7:**
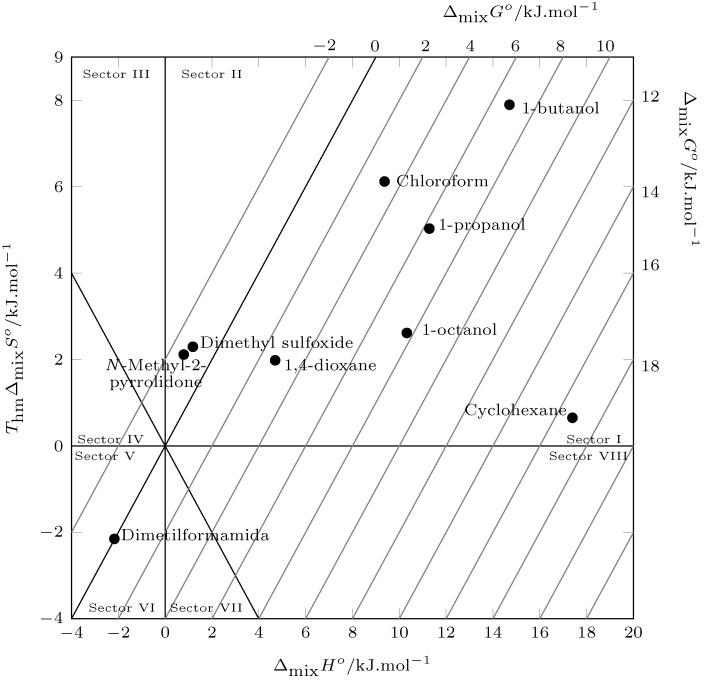
Relation between enthalpy ($\Delta_{\mathrm{mix}}H^{o}$) and entropy ($T_{\mathrm{hm}}\Delta_{\mathrm{mix}}S^{o}$) of the process mixing ofpyrazinamide (3) in nine organic solvents at $T_{\mathrm{hm}}$. The isoenergetic curves for $\Delta_{\mathrm{mix}}G^{o}$ are represented by dotted lines.

According to Perlovich’s analysis (Figure 7), the mixing process is driven by the enthalpy of mixing in the solvents 1-propanol, 1-butanol, 1-octanol, chloroform, 1,4-dioxane, and cyclohexane (Sector I: $\Delta_{\mathrm{mix}}H^{o}>T\Delta_{\mathrm{mix}}S^{o}$), and in the solvents *N*-Methyl-2-pyrrolidone, dimethylformamide, and dimethyl sulfoxide, the mixing process is driven by the mixing entropy. (Sector II: $T\Delta_{\mathrm{mix}}S^{o}>\Delta_{\mathrm{mix}}H^{o}$ and Sector VI: $\Delta_{\mathrm{mix}}H^{o}<0;T\Delta_{\mathrm{mix}}S^{o}<0;|T\Delta_{\mathrm{mix}}S^{o}\left|>\right|\Delta_{\mathrm{mix}}H^{o}|$, $|\Delta_{\mathrm{mix}}H^{o}|<|T\Delta_{\mathrm{mix}}S^{o}|$ (Figure 7)).

### 3.3. Solubility Parameters of Pyrazinamide

Rivas-Ozuna et al. [31], reported the solubility parameter of pyrazinamide calculated according to the Fedor method as 35.10 MPa^1/2^.

Another method for calculating the solubility parameter by group contribution is the Hoftyzer-van Krevelen method [56], in accordance with:

(8) $$\delta_{T}=\sqrt{{\left[\sum nF_{d}\cdot V^{-1}\right]}^{2}+{\left[\sqrt{\sum n{\left(F_{p}\right)}^{2}}\cdot V^{-1}\right]}^{2}+{\left[\sqrt{\sum nF_{h}\cdot V^{-1}}\right]}^{2}}$$

where $F_{d}$ (in ${\left(J\cdot {\mathrm{cm}}^{3}\right)}^{1/2}\cdot {\mathrm{mol}}^{-1}$) represents the contribution of dispersion forces, $F_{p}$ (in $J\cdot {\mathrm{cm}}^{3}\cdot {\mathrm{mol}}^{-2}$) represents the contribution of polarity strength, $F_{h}$ (in $J\cdot {\mathrm{mol}}^{-1}$) represents the contribution of the hydrogen bond interaction energy, and *V* is the molar volume of the drug, calculated by Fedors’ method (in ${\mathrm{cm}}^{3}\cdot {\mathrm{mol}}^{-1}$) [32].

Table 5 shows the calculation of the solubility parameter of pyrazinamide according to Hoftyzer-van Krevelen. In this case, the result is 32.90 MPa^1/2^, which is 6% lower than the result obtained using Fedors’ method.

Another method to calculate the solubility parameter of a drug was reported by Bustamante et al. [59,60]. This method, unlike the group contribution methods of Fedors and Hoftyzer-van Krevelen, uses experimental data of drug solubility in different pure solvents, correlated the logarithm of the solubility according to the following expression:

(9) $$\ln x_{2}=C_{0}+C_{1}\delta_{d}^{2}+C_{2}\delta_{d}+C_{3}\delta_{p}^{2}+C_{4}\delta_{p}+C_{5}\delta_{h}^{2}+C_{6}\delta_{h}$$

Once the coefficients of Equation (9) are obtained, the solubility parameters $\delta_{d}$, $\delta_{p}$ and $\delta_{h}$ of the drug are obtained as:

(10) $$\delta_{d}=-\frac{C_{2}}{2C_{1}}$$

(11) $$\delta_{P}=-\frac{C_{4}}{2C_{3}}$$

(12) $$\delta_{h}=-\frac{C_{6}}{2C_{5}}$$

(13) $$\delta_{T}=\sqrt{\delta_{d}^{2}+\delta_{P}^{2}+\delta_{h}}$$

Table 6 shows the partial parameters that represent the dispersion ($\delta_{d}$), polar ($\delta_{p}$), and hydrogen bonding ($\delta_{h}$) components of the total solubility parameter ($\delta_{T}$) of some solvents and the solubility data of pyrazinamide.

Therefore, the parameters of Equation (9) are calculated as:

(14) $$\begin{array}{c} \ln x_{2}=-5.733\pm 74.371+0.072\pm 0.255\delta_{d}^{2}-1.421\pm 8.708\delta_{d}+0.015\pm 0.020\delta_{p}^{2}\\ -0.138\pm 0.389\delta_{p}-0.007\pm 0.003\delta_{h}^{2}+0.384\pm 0.180\delta_{h} \end{array}$$

(15) $$\delta_{d}=-\left(\frac{-1.421\;{\mathrm{MPa}}^{-1/2}}{2\cdot 0.072\;{\mathrm{MPa}}^{-1}}\right)=9.855\;{\mathrm{MPa}}^{1/2}$$

(16) $$\delta_{P}=-\left(\frac{-0.138\;{\mathrm{MPa}}^{-1/2}}{2\cdot 0.015\;{\mathrm{MPa}}^{-1}}\right)=4.621\;{\mathrm{MPa}}^{1/2}$$

(17) $$\delta_{h}=-\left(\frac{0.384\;{\mathrm{MPa}}^{-1/2}}{2\cdot -0.007\;{\mathrm{MPa}}^{-1}}\right)=25.954\;{\mathrm{MPa}}^{1/2}$$

(18) $$\delta_{T}=\sqrt{\left(9.855^{2}+4.621^{2}+25.954^{2}\right)\;\mathrm{MPa}}=28.14\;{\mathrm{MPa}}^{1/2}$$

From the parameters of Equation (14), operating on Equations (15)–(18), the solubility parameter of pyrazinamide is calculated as 28.14 MPa^1/2^

When comparing the result with the experimental values found by Rivas-Ozuna et al. (points of maximum solubility in cosolvent mixtures), in the mixtures 1,4-dioxane + Water (24.7 MPa^1/2^ [31]) and ethanol + water (31.9 MPa^1/2^ [31]), the difference is between 12–14%. When comparing them with the values obtained by the Fedors method (35.1 MPa^1/2^ [31]) and the Hoftyzer-van Krevelen method (32.9 MPa^1/2^), the difference is in the range of 14–20%. This variation in the results is very common, since the group contribution methods are tools that sometimes allow good approximations to be made but do not take into account some molecular interactions that can be observed by Rivas-Ozuna et al. [31], and some studies conducted by Bustamante et al., where similar behaviors have been reported [61].

## 4. Materials and Methods

### 4.1. Reagents

All the reagents used in the research are reported in Table 7. Some relevant information regarding the quality of each of the reagents is specified.

### 4.2. Solubility Determination

Like other research published by our group, the solubility of pyrazinamide solubility was determined according to the shake-flask method proposed by Higuchi and Connors [62,63,64]. The method is described in detail in some open access publications [65].

Overall, the method consists of 5 steps.

1. Saturation of the solvent: In an amber colored bottle, 5.0 mL of solvent is added; then, pyrazinamide is added with vigorous stirring until a saturated solution is obtained (this process is verified by measuring the concentration of the drug until a constant concentration is obtained).
2. Thermodynamic equilibrium: To ensure solvent saturation, the samples remain for 36 h at constant temperature (at each of the study temperatures) in a recirculation bath (Medingen K-22/T100, Medingen, Germany). To ensure thermodynamic equilibrium, in all cases, a sufficient amount of pyrazinamide is added to generate an equilibrium between the saturated solution and a quantity of undissolved solid drug (usually remaining at the bottom of the flask).
3. Filtration: To ensure that no undissolved solids are taken up at the time of quantification, the samples are filtered through 0.45 μm membranes (Millipore Corp. Swinnex-13, Burlington, MA, USA).
4. Quantification: The method used is UV/Vis spectrometry; thus, the wavelength of maximum absorbance of pyrazinamide (267 nm ($\lambda_{\max}$)) is determined and a calibration curve is designed in the range of compliance with the Lambert–Beer law (UV/Vis EMC-11- UV spectrophotometer, Duisburg, Germany). For solutions with very low concentrations, the standard addition method described by Caviedes-Rubio et al. [66] is used.
5. Evaluation of the solid phase: To evaluate possible polymorphic changes or decomposition of pyrazinamide, the solid phases in equilibrium with the saturated solutions are analyzed by DSC. The solution is first saturated at a temperature higher than the study temperature so that the drug portion precipitates. This precipitate is collected, dried, and subjected to DSC analysis to determine whether polymorphic changes have occurred with respect to the original sample.

### 4.3. Calorimetric Study

The enthalpy and melting temperature of 10 pyrazinamide samples were determined by DSC (DSC 204 F1 Phoenix, Dresden, Germany). The equipment was calibrated using Indium and Tin as standards and an empty sealed pan was used as reference. A mass of approximately 10.0 mg of each sample was deposited in an aluminum crucible and placed in the calorimeter under a nitrogen flow of 10 mL·min^−1^. The heating cycle was developed from 380 to 500 K, with a heating ramp of 10 K·min^−1^. The solid samples in equilibrium with the saturated solution were dried at room temperature for 48 h under a continuous stream of dry air [66].

## 5. Conclusions

The solubility of pyrazinamide increases as the temperature of the system increases, indicating an endothermic process, favored by the entropy of solution (positive values); however, the enthalpy of solution is the thermodynamic function of greater energetic contribution to the solution process, being the thermodynamic function that drives it. When evaluating the mixing process, DMF, DMSO, and NMP are the solvents that most favor the mixing process.

Bearing in mind that the solubility parameter is a relevant data point when evaluating possible means to improve solubility (dielectric requirement) or to decrease it as in crystallization processes, the solubility parameter was determined by the Hoftyzer-van Krevelen and Bustamante methods, presenting differences of up to 20%, which indicates the relevance of the experimental data.

## Figures and Tables

**Table 1:** Experimental solubility of pyrazinamide (2) in some organic solvents expressed in mole fraction ($x_{2}$ ± standard deviations) at different temperatures. Experimental pressure *p*: 0.096 MPa *^a^*.

Solvent	Temperature/K *^b^*				
	278.15	283.15	288.15	293.15	298.15
Cyclohexane *^c^*		1.73 ± 0.05	2.48 ± 0.05	3.302 ± 0.026	4.21 ± 0.08
1,4-dioxane *^d^*			8.42 ± 0.20	9.25 ± 0.09	11.58 ± 0.23
Chloroform *^d^*	3.83 ± 0.26	5.24 ± 0.08	6.8 ± 0.1	8.47 ± 0.07	9.85 ± 0.27
DMF *^d^*	21.8 ± 0.6	25.13 ± 0.29	28.4 ± 0.4	31.5 ± 0.7	35.5 ± 0.5
DMSO *^d^*				46.6 ± 0.4	50.2 ± 1.2
NMP *^d^*	1.19 ± 0.07	1.14 ± 0.03	1.16 ± 0.10	1.82 ± 0.16	2.13 ± 0.12
1-propanol *^d^*	1.167 ± 0.033	1.532 ± 0.03	1.83 ± 0.04	2.29 ± 0.05	2.9 ± 0.05
1-butanol *^d^*	0.923 ± 0.03	1.089 ± 0.01	1.41 ± 0.04	1.75 ± 0.04	2.2 ± 0.022
1-octanol *^d^*	0.717 ± 0.017	0.849 ± 0.015	1.052 ± 0.012	1.235 ± 0.019	1.49 ± 0.031
Solvent	Temperature/K *^b^*				
	303.15	308.15	313.15	318.15	
Cyclohexane *^c^*	5.15 ± 0.13	6.34 ± 0.09	7.42 ± 0.21	8.59 ± 0.14	
1,4-dioxane *^d^*	12.99 ± 0.18	14.8 ± 0.4	16.5 ± 0.4	19.3 ± 0.4	
Chloroform *^d^*	11.56 ± 0.15	12.65 ± 0.27	14.64 ± 0.32	16.00 ± 0.18	
DMF *^d^*	37.6 ± 0.7	41.9 ± 1.4	44.0 ± 1.0	46.8 ± 0.9	
DMSO *^d^*	58.8 ± 1.0	66.4 ± 2.0	72.4 ± 0.5	81.6 ± 0.4	
NMP *^d^*	2.34 ± 0.09	2.59 ± 0.09	2.83 ± 0.14	2.99 ± 0.09	
1-propanol *^d^*	3.403 ± 0.025	3.99 ± 0.08	4.61 ± 0.09	5.11 ± 0.17	
1-butanol *^d^*	2.73 ± 0.10	3.26 ± 0.06	4.09 ± 0.10	4.69 ± 0.13	
1-octanol *^d^*	1.818 ± 0.026	2.21 ± 0.033	2.56 ± 0.08	2.93 ± 0.03	

*^a^* Standard uncertainty in pressure *u(p)* = 0.001 MPa. *^b^* Standard uncertainty in temperature is *u(T)* = 0.05 K. *^c^* 10^5^*x*_2_. *^d^* 10^3^*x*_2_.

**Table 2:** The thermophysical properties of pyrazinamide obtained by the DSC.

Sample	Polymorphic Transition		Fusion		Ref.
	$T_{\mathrm{trs}}$/K	$\Delta_{\mathrm{trs}}H$/kJ·mol^−1^	$T_{\mathrm{fus}}$/K	$\Delta_{\mathrm{fus}}H$/kJ·mol^−1^	
Original sample	422.0±0.5	1.70±0.5	463.9±0.5	25.3±0.5	[31]
	422.0	1.63	463.0	27.41	[42]
	420.05	1.63	461.45	28.10	[43]
	420.65	1.40	461.55	24.71	[44]
	421.11	1.52	462.11	25.47	[33]
Cyclohexane	−	−	463.2±0.5	25.5±0.5	This work
1,4-dioxane	−	−	463.5±0.5	26.1±0.5	This work
Chloroform	−	−	461.9±0.5	25.8±0.5	This work
DMF	−	−	464.2±0.5	26.2±0.5	This work
DMSO	−	−	463.7±0.5	25.7±0.5	This work
NMP	−	−	464.3±0.5	25.8±0.5	This work
1-propanol	−	−	462.8±0.5	26.0±0.5	This work
1-butanol	−	−	463.1±0.5	26.3±0.5	This work
1-octanol	−	−	463.4±0.5	25.4±0.5	This work
Form α	428.25	1.3	461.1	26.1	[41]
Form β(+γ)	372.55	0.78	461.2	25.5	[41]
Form γ	−	−	461.1	26.5	[41]
Form δ	404.355	1.66	461.95	26.9	[41]

**Table 3:** Thermodynamic functions of the solution process of pyrazinamide (2), some organic solvents at $T_{\mathrm{hm}}$, and pressure *p* = 0.096 MPa *^a^*.

Solvent	$\Delta_{\mathrm{soln}}G^{o}$/kJ·mol^−1^	$\Delta_{\mathrm{soln}}H^{o}$/kJ·mol^−1^	$T\Delta_{\mathrm{soln}}S^{o}$/kJ·mol^−1^
Cyclohexane *^b^*	25.08 ± 0.06	33.8 ± 1.5	8.7 ± 1.5
1,4-dioxane *^c^*	10.980 ± 0.027	21.2 ± 0.8	10.2 ± 0.8
Chloroform *^d^*	11.65 ± 0.07	25.6 ± 1.6	13.9 ± 1.6
DMF *^d^*	8.387 ± 0.024	14.0 ± 0.6	5.7 ± 0.6
DMSO *^e^*	7.083 ± 0.021	17.8 ± 0.8	10.8 ± 0.8
NMP *^d^*	15.35 ± 0.03	17.0 ± 0.6	1.7 ± 0.6
1-propanol *^d^*	14.66 ± 0.04	27.5 ± 0.8	12.8 ± 0.8
1-butanol *^d^*	15.212 ± 0.02	30.9 ± 0.5	15.7 ± 0.5
1-octanol *^d^*	16.11 ± 0.016	26.5 ± 0.4	10.4 ± 0.4

*^a^* Local atmospheric pressure. *^b^* $T_{\mathrm{hm}}=300.2$ K. *^c^* $T_{\mathrm{hm}}=302.8$ K. *^d^* $T_{\mathrm{hm}}=297.6$ K. *^e^* $T_{\mathrm{hm}}=305.4$ K.

**Table 4:** Thermodynamic functions of mixing pyrazinamide (2) with some organic solvents at $T_{\mathrm{hm}}$ and pressure *p* = 0.096 MPa *^a^*.

Solvent	$\Delta_{\mathrm{mix}}G^{o}$/kJ·mol^−1^	$\Delta_{\mathrm{mix}}H^{o}$/kJ·mol^−1^	$T\Delta_{\mathrm{mix}}S^{o}$/kJ·mol^−1^
cyclohexane *^b^*	16.74 ± 0.08	17.4 ± 1.6	0.7 ± 1.6
1,4-dioxane *^c^*	2.72 ± 0.06	4.7 ± 1	2.0 ± 1.0
Chloroform *^d^*	3.25 ± 0.08	9.4 ± 1.7	6.1 ± 1.7
DMF *^d^*	−0.02 ± 0.06	−2.2 ± 0.8	−2.2 ± 0.8
DMSO *^e^*	−1.11 ± 0.05	1.2 ± 0.9	2.3 ± 0.9
NMP *^d^*	−1.32 ± 0.06	0.8 ± 0.8	2.1 ± 0.8
1-propanol *^d^*	6.25 ± 0.06	11.3 ± 1	5.0 ± 1.0
1-butanol *^d^*	6.81 ± 0.05	14.7 ± 0.7	7.9 ± 0.7
1-octanol *^d^*	7.71 ± 0.05	10.3 ± 0.6	2.6 ± 0.6

*^a^* Local atmospheric pressure. *^b^* $T_{\mathrm{hm}}=300.2$ K. *^c^* $T_{\mathrm{hm}}=302.8$ K. *^d^* $T_{\mathrm{hm}}=297.6$ K. *^e^* $T_{\mathrm{hm}}=305.4$ K.

**Table 5:** Hoftyzer-van Krevelen methods applied to calculate partial and total solubility parameters of pyrazinamide.

Group	n	Hoftyzer-van Krevelen Parameters		
		$F_{d}$	$F_{p}^{2}$	$F_{h}$
−CH=	3	$3\cdot 200^{a}=600$	0 *^a^*	0*^a^*
>C=	1	70 *^a^*	0 *^a^*	0 *^a^*
Ring	1	190 *^a^*	0 *^b^*	0 *^b^*
−N=	2	$2\cdot 20^{a}=40$	$2\cdot 800^{2(a)}=1.28\cdot 10^{6}$	$2\cdot 5000^{a}$ = 10,000
−CO−	1	290 *^a^*	$770^{2(a)}=59.29\cdot 10^{4}$	2000 *^a^*
−NH_2_	1	280 *^a^*	$310^{2(b)}=96.1\cdot 10^{3}$	8400 *^a^*
	∑	1470	1,969,000	15,400 *^a^*
	δ (MPa^1/2^)	1470/71.9=20.44	(1,969,000)^1/2^/71.9=19.52	(15,400/71.9)^1/2^ =16.84
$\delta_{T}=\sqrt{20.44^{2}+19.52^{2}+16.84^{2}}=32.90$ MPa^1/2^				

*^a^* from van Krevelen and Hoftyzer [57]. *^b^* from Ravindra et al. [58].

**Table 6:** Logarithmic mole fraction solubility of pyrazinamide (2) and Hansen solubility parameters of solvents at *T* = 298.15 K.

Solvent	Hansen Solubility Parameters *^a^*				$\ln x_{3}$
	$\delta_{d}$/MPa^0.5^	$\delta_{p}$ ^0.5^	$\delta_{h}$ ^0.5^	$\delta_{T}$ ^0.5^	
Cyclohexane	16.8	0.0	0.2	16.8	−9.943
Chloroform	17.8	3.1	5.7	18.9	−4.620
1,4-dioxane	19.0	1.8	7.4	20.5	−4.458
Acetonitrile	15.3	18.0	6.1	24.4	−5.831 *^b^*
Water	15.6	16.0	42.3	47.8	−5.644 *^c^*
DMF	17.4	13.7	11.3	24.9	−3.337
DMSO	18.4	16.4	10.2	26.7	−2.992
NMP	18.0	12.3	7.2	23.0	−6.153
Methanol	15.1	12.3	22.3	29.6	−5.36 *^b^*
Ethanol	15.8	8.8	19.4	26.5	−5.960 *^c^*
1-propanol	16.0	6.8	17.4	24.6	−5.843
2-propanol	15.8	7.2	16.0	23.6	−6.101 *^b^*
1-butanol	16.0	5.7	15.8	23.2	−6.119
2-butanol	15.8	5.7	14.5	22.2	−6.277 *^b^*
1-octanol	17.0	3.3	11.9	21.0	−6.509

*^a^* From Barton [32]. *^b^* From Zhang et al. [37]. *^c^* From Rivas-Ozuna et al. [31].

**Table 7:** Source and purities of the compounds used in this research.

Chemical Name	CAS ^*a*^	Purity in Mass Fraction	Analytic Technique *^b^*
pyrazinamide *^c^*	98-96-4	>0.990	HPLC
ethanol *^c^*	64-17-5	0.998	GC
cyclohexane *^d^*	110-82-7	0.998	GC
1,4-dioxane *^d^*	123-91-1	0.998	GC
chloroform *^d^*	67-66-3	0.998	GC
*N*,*N*-dimethylformamide (DMF) *^d^*	68-12-2	0.998	GC
dimethyl sulfoxide (DMSO) *^d^*	67-68-5	0.998	GC
*N*-Methyl-2-pyrrolidone (NMP) *^d^*	872-50-4	0.998	GC
1-propanol *^e^*	71-23-8	0.998	GC
1-butanol *^e^*	71-36-3	0.998	GC
1-octanol *^e^*	111-87-5	0.998	GC

*^a^* Chemical Abstracts Service Registry Number. *^b^* HPLC is high-performance liquid chromatography; GC is gas chromatography. *^c^* Sigma-Aldrich, Burlington, MA, USA. *^d^* Supelco, Burlington, MA, USA. *^e^* Merck, Burlington, MA, USA.

## Data Availability

Data is contained within the article or Supplementary Materials.

## Supplementary Materials

The following supporting information can be downloaded at: https://www.mdpi.com/article/10.3390/molecules29215089/s1, Table S1: Ideal solubility of pyrazinamide at different temperatures; Table S2: Thermodynamic functions of the ideal process of pyrazinamide; Figure S1: van’t Hoff plot for ideal solubility data of pyrazinamide using the harmonic mean temperature of the study temperatures.

## References

[1] Suárez I., Fünger S.M., Kröger S., Rademacher J., Fätkenheuer G., Rybniker J. (2019). The diagnosis and treatment of tuberculosis. Dtsch Arztebl Int..

[2] World Health Organization (WHO) (2010). Treatment of Tuberculosis: Guidelines.

[3] Sosa E.J., Burguener G., Lanzarotti E., Defelipe L., Radusky L., Pardo A.M., Marti M., Turjanski A.G., Fernández Do Porto D. (2017). Target-Pathogen: A structural bioinformatic approach to prioritize drug targets in pathogens. Nucleic Acids Res..

[4] Jones N.T., Abadie R., Keller C.L., Jones K., Ledet L.F., Fox J.E., Klapper V.G., Potharaju P., Siddaiah H., Kaye A.M. (2024). Treatment and toxicity considerations in tuberculosis: A narrative review. Cureus.

[5] Delgado D.R. (2024). LaTeX Code of the Chemical Structure of Some Drugs.

[6] NORMAN Network (2023). Emerging Substances. https://www.norman-network.com/nds/susdat/susdatSearchShow.php.

[7] Rubio D.I.C., Camacho Feria D.M., Delgado D.R. (2017). Tratamientos para la remoción de antibacteriales y agentes antimicrobiales presentes en aguas residuales. Rev. Logos Cienc. Tecnol..

[8] Harrower J., McNaughtan M., Hunter C., Hough R., Zhang Z., Helwig K. (2021). Chemical fate and partitioning behavior of antibiotics in the aquatic environment—A review. Environ. Toxicol. Chem..

[9] Yalkowsky S.H. (1999). Solubility and Solubilization in Aqueous Media.

[10] Sinko P.J. (2011). Martin’s Physical Pharmacy and Pharmaceutical Sciences.

[11] Cristancho D.M., Delgado D.R., Martínez F. (2013). Meloxicam solubility in ethanol+ water mixtures according to the extended Hildebrand solubility approach. J. Solut. Chem..

[12] Jouyban A., Fakhree M.A.A., Shayanfar A. (2010). Review of pharmaceutical applications of *N*-methyl-2-pyrrolidone. J. Pharm. Pharm. Sci..

[13] Liu X., Abraham M.H., Acree W.E. (2022). Abraham model descriptors for melatonin; prediction of Solution, biological and thermodynamic properties. J. Solut. Chem..

[14] Llompart P., Minoletti C., Baybekov S., Horvath D., Marcou G., Varnek A. (2024). Will we ever be able to accurately predict solubility?. Sci. Data Vol..

[15] Gupta Soni A., Joshi R., Soni D., Deep Kaur C., Saraf S., Kumar Singh P. (2024). Predicting Drug Properties: Computational Strategies for Solubility and Permeability Rates. Software and Programming Tools in Pharmaceutical Research.

[16] Macedo B., Taveira-Gomes T., Pais A., Vitorino C., Nunes S., Cova T. (2025). Automating drug discovery. Artificial Intelligence for Drug Product Lifecycle Applications.

[17] Yuan H., Yang J., Wang M., Li H., Li Y., Li T., Ren B. (2025). Solubility measurement and data correlation of 2-ethoxy-1-naphthoic acid in twelve pure solvents at temperatures from 278.15 to 323.15 K. J. Chem. Thermodyn..

[18] Sorkun M.C., Koelman J.M.V.A., Er S. (2021). Pushing the limits of solubility prediction via quality-oriented data selection. iScience.

[19] Redlich O., Kwong J.N.S. (1949). On the thermodynamics of solutions. V. an equation of state. Fugacities of gaseous solutions. Chem. Rev..

[20] Yaws C.L., Narasimhan P.K., Lou H.H., Pike R.W. (2005). Solubility of Chemicals in Water. Water Encyclopedia.

[21] Hokkala E., Strachan C.J., Agopov M., Järvinen E., Semjonov K., Heinämäki J., Yliruusi J., Svanbäck S. (2024). Thermodynamic solubility measurement without chemical analysis. Int. J. Pharm..

[22] Sharapova A.V., Ol’khovich M.V., Blokhina S.V. (2024). Thermodynamic consideration of dissolution and distribution behavior of carvedilol in pharmaceutical significant media. J. Chem. Thermodyn..

[23] Lee J.L., Chong G.H., Kanno A., Ota M., Guo H., Smith R.L. (2024). Local composition-regular solution theory for analysis of pharmaceutical solubility in mixed-solvents. J. Mol. Liq..

[24] Jouyban A., Khezri S., Jafari P., Zarghampour A., Acree W.E. (2023). A new set of solute descriptors to calculate solubility of drugs in mono-solvents. Ann. Pharm. Françaises.

[25] Marcus Y. (1998). The Properties of Solvents.

[26] Marcus Y. (1998). Solvent Mixtures: Properties and Selective Solvation.

[27] Marcus Y. (2008). On the preferential solvation of drugs and PAHs in binary solvent mixtures. J. Mol. Liq..

[28] Tayyebi A., Alshami A., Rabiei Z., Yu X., Ismail N., Talukder M., Power J. (2023). Prediction of organic compound aqueous solubility using machine learning: A comparison study of descriptor-based and fingerprints-based models. J Cheminf.

[29] Cuellar-Carmona Y.L., Cerquera N.E., Cardenas-Torres R.E., Ortiz C.P.O., Martínez F., Delgado D.R.D. (2024). Correlation of the solubility of isoniazid in some aqueous cosolvent mixtures using different mathematical models. Braz. J. Chem. Eng..

[30] Hildebrand J.H. (1929). Solubility. XII. Regular solutions. J. Am. Chem. Soc..

[31] Rivas-Ozuna D.A., Ortiz C.P., Delgado D.R., Martínez F. (2024). Solubility and preferential solvation of pyrazinamide in some aqueous-cosolvent mixtures at 298.15 K. Int. J. Thermophys..

[32] Barton A.F.M. (1991). Handbook of Solubility Parameters and Other Cohesion Parameters.

[33] Maharana A., Sarkar D. (2019). Solubility measurements and thermodynamic modeling of pyrazinamide in five different solvent-antisolvent mixtures. Fluid Phase Equilibria.

[34] Hermanto M.W., Yeoh A., Soh B., Chow P.S., Tan R.B. (2015). Robust crystallization process development for the metastable *δ*-form of pyrazinamide. Org. Process Res. Dev..

[35] Xi S., Wang S., Lu G., Wang J. (2019). Solubility measurement, correlation and cosolvent phenomena for pyrazinamide in two mixed solvents. J. Beijing Univ. Chem. Technol. (Nat. Sci.).

[36] Blokhina S.V., Ol’khovich M.V., Sharapova A.V., Volkova T.V., Perlovich G.L. (2015). Solution thermodynamics of pyrazinamide, isoniazid, and p-aminobenzoic acid in buffers and octanol. J. Chem. Thermodyn..

[37] Zhang K., Shen H., Xu S., Zhang H., Zhu M., Shi P., Fu X., Yang Y., Gong J. (2017). Thermodynamic study of solubility for pyrazinamide in ten solvents from T = (283.15 to 323.15) K. J. Chem. Thermodyn..

[38] Bernstein J. (1989). Polymorphism in drug design and delivery. Prog. Clin. Biol. Res..

[39] Singhal D., Curatolo W. (2004). Drug polymorphism and dosage form design: A practical perspective. Adv. Drug Deliv. Rev..

[40] Grunenberg A., Keil B., Henck J.O. (1995). Polymorphism in binary mixtures, as exemplified by nimodipine. Int. J. Pharm..

[41] Cherukuvada S., Thakuria R., Nangia A. (2010). Pyrazinamide polymorphs: Relative stability and vibrational spectroscopy. Cryst. Growth Des..

[42] Negoro A., Miki T., Ueda S., Sanada T., Okada R. (1960). Solubility phenomena of pyridine- and pyrazine-monocarboxamides. II: Heats of fusion of picolinamide, nicotinamide, isonicotinamide, and pyrazincarboxamide. Yakugaku Zasshi.

[43] Castro R.A.E., Maria T.M.R., Évora A.O.L., Feiteira J.C., Silva M.R., Beja A.M., Canotilho J., Eusébio M.E.S. (2010). A new insight into pyrazinamide polymorphic forms and their thermodynamic relationships. Cryst. Growth Des..

[44] Baaklini G., Dupray V., Coquerel G. (2015). Inhibition of the spontaneous polymorphic transition of pyrazinamide *γ* form at room temperature by co-spray drying with 1,3-dimethylurea. Int. J. Pharm..

[45] Bustamante C., Bustamante P. (1996). Nonlinear enthalpy–entropy compensation for the solubility of phenacetin in dioxane–water solvent mixtures. J. Pharm. Sci..

[46] Peña M.A., Bustamante P., Escalera B., Reíllo A., Bosque-Sendra J.M. (2004). Solubility and phase separation of benzocaine and salicylic acid in 1,4-dioxane–water mixtures at several temperatures. J. Pharm. Biomed. Anal..

[47] Krug R.R., Hunter W.G., Grieger R.A. (1976). Enthalpy-entropy compensation. 1. Some fundamental statistical problems associated with the analysis of van’t Hoff and Arrhenius data. J. Phys. Chem..

[48] Krug R.R., Hunter W.G., Grieger R.A. (1976). Enthalpy-entropy compensation. 2. Separation of the chemical from the statistical effect. J. Phys. Chem..

[49] Delgado D.R., Rodríguez G.A., Martínez F. (2013). Thermodynamic study of the solubility of sulfapyridine in some ethanol+water mixtures. J. Mol. Liq..

[50] Yalkowsky S.H., Wu M. (2010). Estimation of the ideal solubility (crystal-liquid fugacity ratio) of organic compounds. J. Pharm. Sci..

[51] Hildebrand J.H., Prausnitz J.M., Scott R.L. (1970). Regular and Related Solutions: The Solubility of Gases, Liquids, and Solids.

[52] Neau S.H., Flynn G.L. (1990). Solid and liquid heat capacities of n-alkyl para-aminobenzoates near the melting point. Pharm. Res..

[53] Neau S.H., Bhandarkar S.V., Hellmuth E.W. (1997). Differential molar heat capacities to test ideal solubility estimations. Pharm. Res..

[54] Opperhuizen A., Gobas F.A.P.C., Van der Steen J.M.D., Hutzinger O. (1988). Aqueous solubility of polychlorinated biphenyls related to molecular structure. Environ. Sci. Technol..

[55] Ortiz C.P., Cardenas-Torres R.E., Herrera M., Delgado D.R. (2023). Numerical analysis of sulfamerazine solubility in acetonitrile + 1-Propanol cosolvent mixtures at different temperatures. Sustainability.

[56] van Krevelen D., te Nijenhuis K. (2009). Properties of Polymers: Their Correlation with Chemical Structure; Their Numerical Estimation and Prediction from Additive Group Contributions.

[57] van Krevelen D., Hoftyzer P. (1976). Properties of Polymers, Their Estimation and Correlation with Chemical Structure.

[58] Ravindra R., Krovvidi K.R., Khan A. (1998). Solubility parameter of chitin and chitosan. Carbohydr. Polym..

[59] Bustamante P., Martin A., Gonzalez-Guisandez M. (1993). Partial solubility parameters and solvatochromie parameters for predicting the solubility of single and multiple drugs in individual solvents. J. Pharm. Sci..

[60] Peña-Fernandez M.A., Spano G., Torres-Pabón N.S., Martínez F. (2023). Solubility data and solubility parameters of barnidipine in different pure solvents. Ars Pharm..

[61] Bustamante P., Ochoa R., Reillo A., Escalera J.B. (1994). Chameleonic effect of sulfanilamide and sulfamethazine in solvent mixtures. Solubility curves with two maxima. Chem. Pharm. Bull..

[62] Higuchi T., Connors K. (1965). Advances in Analytical Chemistry and Instrumentation.

[63] Dittert L.W., Higuchi T., Reese D.R. (1964). Phase solubility technique in studying the formation of complex salts of triamterene. J. Pharm. Sci..

[64] Mader W.J., Higuchi T. (1970). Phase solubility analysis. CRC Crit. Rev. Anal. Chem..

[65] Ortiz C.P., Cardenas-Torres R.E., Herrera M., Delgado D.R. (2023). Thermodynamic analysis of the solubility of propylparaben in acetonitrile + water cosolvent mixtures. Sustainability.

[66] Caviedes-Rubio D.I., Ortiz C.P., Martinez F., Delgado D.R. (2023). Thermodynamic assessment of triclocarban dissolution process in N-Methyl-2-pyrrolidone + water cosolvent mixtures. Molecules.

