# Supplementary material for: Thermodynamic Assessment of the Pyrazinamide Dissolution Process in Some Organic Solvents
Source: Molecules. 2024 Oct 28;29(21):5089. doi: 10.3390/molecules29215089 (PMC11547866; doi:10.3390/molecules29215089)
Supplement: Supplementary file 1 [file molecules-29-05089-s001.zip › molecules-3233686-supplementary.pdf]

Thermodynamic assessment of the pyrazinamide dissolution process in some organic solvents

Jesus Tovar-Amézquita, Cristian Rincón-Guio, Francy Elaine Torres-Suarez, Magda Melissa Florez, Claudia Patricia Ortiz, Fleming Martinez and Daniel Ricardo Delgado

To calculate the ideal solubility, the temperature and enthalpy of transition and the temperature and enthalpy of melting of the commercial sample are taken.

- T_{trs}=422.0 K
- $\Delta_{\text{trs}}H$= 1.70 kJ·mol^{-1}
- T_{fus}=463.9 K
- $\Delta_{\text{fus}}H$= 25.3 kJ·mol^{-1}

Table S1: Ideal solubility of pyrazinamide at different temperatures

T (K)	278.15	283.15	288.15	293.15	298.15	303.15	308.15	313.15	318.15
$\ln x_2$	−3.36	−3.24	−3.12	−3.01	−2.90	−2.79	−2.68	−2.58	−2.47
x_2	0.0349	0.0392	0.0440	0.0493	0.0550	0.0614	0.0683	0.0759	0.0843

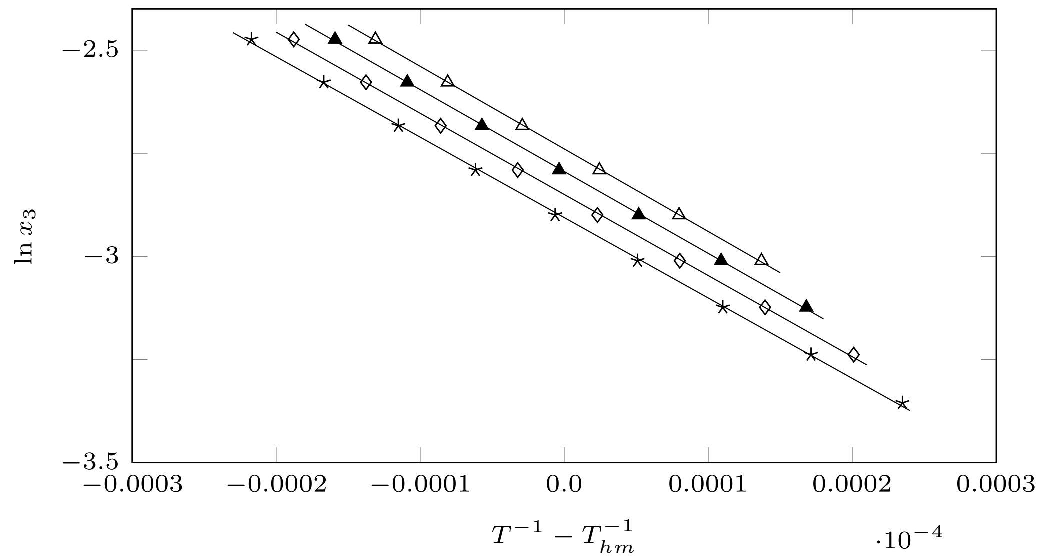

Figure S1: van't Hoff plot for ideal solubility data of pyrazinamide using the harmonic mean temperature of the study temperatures. △ : T_{hm}: 305.4; ▲ : T_{hm}: 302.8; ◊ : T_{hm}: 300.2; ⋆ : T_{hm}: 297.5;

The calculations of the thermodynamic functions of the ideal process were performed according to equations 1-4

Table S2: Thermodynamic functions of the ideal process of pyrazinamide

T_{hm}(K)	$\Delta_{\mathrm{id}}G$/kJ·mol^{-1}	$\Delta_{\mathrm{id}}H$/kJ·mol^{-1}	$T\Delta_{\mathrm{id}}S$/kJ·mol^{-1}
297.6	8.404	16.213	7.809
300.2	8.334	16.360	8.025
302.8	8.264	16.505	8.241
305.4	8.193	16.650	8.457
